# Surveys of measles vaccination coverage in eastern and southern Africa: a review of quality and methods used

**DOI:** 10.2471/BLT.14.146050

**Published:** 2015-03-10

**Authors:** Reinhard Kaiser, Messeret E Shibeshi, Jethro M Chakauya, Emelda Dzeka, Balcha G Masresha, Fussum Daniel, Nestor Shivute

**Affiliations:** a Immunization, Vaccines and Emergencies, WHO Regional Office for Africa, Inter-country Support Team for East and Southern Africa, 86 Enterprise Road, Highlands, Harare, Zimbabwe.; b Immunization, Vaccines and Emergencies, WHO Regional Office for Africa, Brazzaville, Congo.

## Abstract

**Objective:**

To assess the methods used in the evaluation of measles vaccination coverage, identify quality concerns and provide recommendations for improvement.

**Methods:**

We reviewed surveys that were conducted to evaluate supplementary measles immunization activities in eastern and southern Africa during 2012 and 2013. We investigated the organization(s) undertaking each survey, survey design, sample size, the numbers of study clusters and children per study cluster, recording of immunizations and methods of analysis. We documented sampling methods at the level of clusters, households and individual children. We also assessed the length of training for field teams at national and regional levels, the composition of teams and the supervision provided.

**Findings:**

The surveys were conducted in Comoros, Eritrea, Ethiopia, Kenya, Lesotho, Malawi, Mozambique, Namibia, Rwanda, Swaziland, Uganda, Zambia and Zimbabwe. Of the 13 reports we reviewed, there were weaknesses in 10 of them for ethical clearance, 9 for sample size calculation, 6 for sampling methods, 12 for training structures, 13 for supervision structures and 11 for data analysis.

**Conclusion:**

We recommend improvements in the documentation of routine and supplementary immunization, via home-based vaccination cards or other records. For surveys conducted after supplementary immunization, a standard protocol is required. Finally, we recommend that standards be developed for report templates and for the technical review of protocols and reports. This would ensure that the results of vaccination coverage surveys are accurate, comparable, reliable and valuable for programme improvement.

## Résumé

**Objectif:**

Évaluer les méthodes employées dans l'évaluation de la couverture vaccinale antirougeoleuse, identifier les problèmes de qualité et formuler des recommandations d'amélioration.

**Méthodes:**

Nous avons passé en revue les enquêtes d'évaluation des activités supplémentaires de vaccination antirougeoleuse menées en 2012 et 2013 dans des pays de l'est et du sud de l'Afrique. Nous avons spécifiquement étudié la (ou les) structure(s) chargée(s) de mener chaque enquête, la conception des enquêtes (sondages par grappes), la taille des échantillons, le nombre de grappes étudiées et d'enfants par grappe, l'enregistrement des vaccinations et les méthodes d'analyse. Nous avons documenté les méthodes d'échantillonnage appliquées au niveau des grappes, des ménages et des enfants considérés individuellement. Nous avons également évalué la durée de la formation des équipes de terrain, aux niveaux nationaux et régionaux, la composition de ces équipes et les activités de supervision qui ont été réalisées.

**Résultats:**

Ces enquêtes ont été menées aux Comores, en Érythrée, en Éthiopie, au Kenya, au Lesotho, au Malawi, au Mozambique, en Namibie, en Ouganda, au Rwanda, au Swaziland, en Zambie et au Zimbabwe. Sur les 13 rapports que nous avons étudiés, des lacunes ont été constatées concernant les autorisations par comité d'éthique (10 rapports), le calcul de la taille des échantillons (9 rapports), les méthodes d'échantillonnage (6 rapports), les activités de formation (12 rapports), les structures de supervision (13 rapports) et l'analyse des données (11 rapports).

**Conclusion:**

Nous recommandons des améliorations dans la documentation des campagnes de vaccination de routine et des activités de vaccination supplémentaires, au moyen de cartes de vaccination conservées au domicile ou d'autres types de documents. Pour les enquêtes faisant suite à des activités de vaccination supplémentaires, un protocole standard doit être défini. Enfin, nous recommandons d'établir des normes pour la rédaction des rapports et pour l'examen technique des protocoles et des rapports. Ainsi, nous pourrions obtenir des résultats d'enquêtes de couverture vaccinale précis, comparables, fiables et directement exploitables en vue de l'amélioration des programmes.

## Resumen

**Objetivo:**

Evaluar los métodos utilizados en la evaluación de la cobertura de vacunación antisarampionosa, identificar las preocupaciones en materia de calidad y proporcionar recomendaciones de mejora.

**Métodos:**

Se llevó a cabo una revisión de las encuestas que se realizaron para evaluar las actividades suplementarias de inmunización antisarampionosa en el África oriental y meridional durante 2012 y 2013. Se investigaron las organizaciones que realizaron las encuestas, el diseño de las encuestas, el tamaño de las muestras, la cantidad de grupos de estudio y los niños en cada grupo, los registros de inmunizaciones y los métodos de análisis. Se documentaron los métodos de muestreo a nivel de los grupos, de los hogares y de los niños a nivel individual. También se evaluó la duración de la formación para los equipos sobre el terreno a nivel nacional y regional, la composición de los equipos y la supervisión proporcionada.

**Resultados:**

Las encuestas se llevaron a cabo en Comoras, Eritrea, Etiopía, Kenya, Lesotho, Malawi, Mozambique, Namibia, Rwanda, Swazilandia, Uganda, Zambia y Zimbabwe. De los 13 informes que se revisaron, se observaron deficiencias en 10 de ellos por aprobación ética, en 9 por el cálculo del tamaño de las muestras, en 6 por los métodos de muestreo, en 12 por las estructuras de formación, en 13 por las estructuras de supervisión y en 11 por el análisis de los datos.

**Conclusión:**

Se recomienda realizar mejoras en la documentación de la inmunización rutinaria y suplementaria, a través de tarjetas de vacunación en el domicilio y otros registros. Para las encuestas realizadas después de la inmunización suplementaria, se requiere un protocolo normalizado. Finalmente, se recomienda que se elaboren normas para las plantillas de informes y para la revisión técnica de protocolos e informes. Esto garantizaría que los resultados de las encuestas sobre la cobertura de vacunación fuesen precisos, comparables, fiables y valiosos para la mejora del programa.

## ملخص

**الغرض:**

تحديد مدى فعالية الطرائق المتبعة لتقييم التغطية بخدمات التمنيع ضد الحصبة، والوقوف على الشواغل المرتبطة بالجودة النوعية، وتقديم التوصيات الهادفة للتحسّن.

**الطريقة:**

لقد راجعنا المسوح التي تم إجراؤها لتقييم الأنشطة التكميلية للتمنيع ضد الحصبة في شرق أفريقيا وجنوبها خلال عاميّ 2012 و2013. وقد تحرينا عن المؤسسة المسؤولة عن إجراء كل مسح (واحدةً كانت أو أكثر)، وتصميم المسح، وحجم العينة، وأعداد العناقيد المشاركة في الدراسة فضلاً عن عدد الأطفال المشاركين في كل عنقود، حيث قمنا بتسجيل التمنيعات وطرائق التحليل. كما قمنا بتوثيق طرائق الاعتيان على مستوى العناقيد والأُسر والأطفال المنفردين. وقمنا أيضًا بتقييم طول فترة التدريب المقدّم للفرق الميدانية على المستويين الوطني والإقليمي، وتكوين الفِرق، والإشراف المُقدم. ****

**النتائج:**

تم إجراء المسوح في جزر القمر وإريتريا وإثيوبيا وليسوتو وكينيا وملاوي وموزامبيق وناميبيا ورواندا وسوازيلند وأوغندا وزامبيا وزمبابوي. ومن بين التقارير التي خضعت للمراجعة والبالغ عددها 13 تقريرًا، فقد وُجدت مواضع ضعف في 10 منها بشأن التصريح الأخلاقي، و9 بشأن حساب حجم العينة، و6 بشأن طرائق الاعتيان، و12 بشأن هياكل التدريب، و13 بشأن هياكل الإشراف، و11 بشأن تحليل البيانات.

**الاستنتاج:**

إننا نوصي بإدخال تحسينات على توثيق عمليات التمنيع الروتيني والتكميلي، من خلال بطاقات التمنيع المنزلية أو غيرها من السجلات. كما يلزم وضع برتوكول معياري للمسوح التي يتم إجراؤها بعد التمنيع التكميلي. ونوصي في النهاية بوضع معايير لنماذج التقارير والمراجعة التقنية للبروتوكولات والتقارير، حيث سيضمن ذلك دقة نتائج المسوح التي تجري بشأن التغطية بخدمات التمنيع، وإمكانية إخضاعها للمقارنات، والتعويل عليها، واستخلاص القيمة من خلالها لإدخال التحسينات على البرنامج.

## 摘要

**目的:**

旨在评价在评估麻疹疫苗接种覆盖率时采用的方法、确定质量问题并提供改进建议。

**方法:**

我们评审了于 2012 年和 2013 年期间在非洲东部和南部为评估补充麻疹免疫接种活动而开展的调查。我们研究了进行每项调查的组织、调查设计、样本容量、研究群体的人数和每个研究群体中儿童的数量，记录了免疫接种的情况和分析方法。我们把在群体、家庭和个别儿童层面上采用的抽样方法记录在案。我们还评价了现场团队在国家和区域层面的培训时长、团队构成和提供的监管情况。

**结果:**

调查开展地包括科摩罗、厄立特里亚、埃塞俄比亚、莱索托、肯尼亚、马拉维、莫桑比克、纳米比亚、卢旺达、斯威士兰、乌干达、赞比亚和津巴布韦。在我们评审的 13 份报告中，其中 10 份在伦理审批方面有不足之处，9 份在样本容量计算方面有不足之处，6 份在抽样方法方面有不足之处，12 份在培训结构方面有不足之处，13 份在监管结构方面有不足之处，11 份在数据分析方面有不足之处。

**结论:**

我们建议通过家庭疫苗接种卡和其他记录对常规和补充免疫接种文件进行改进。补充免疫接种后开展的调查需采用标准方案。最后，我们建议针对报告模板以及方案和报告的技术评审制定出标准。这将确保疫苗接种覆盖率调查的结果精确可靠，具有可比性，同时有益于改进方案。

## Резюме

**Цель:**

Оценить методы, используемые для оценки охвата вакцинацией против кори, выявить проблемы, связанные с качеством, и предоставить рекомендации по улучшению.

**Методы:**

Мы проанализировали исследования, которые проводились с целью оценки дополнительных мероприятий по иммунизации против кори в восточной и южной Африке в течение 2012 и 2013 гг. Мы изучили организации, проводившие эти исследования, дизайн исследования, размер выборки, число исследовательских кластеров и детей на один исследовательский кластер, данные по прививкам и методы анализа. Мы задокументировали методы формирования выборки на уровне кластеров, семейных единиц и отдельных детей. Мы также оценили продолжительность обучения полевых групп на национальном и региональном уровнях, состав групп и контроль.

**Результаты:**

Исследование проводилось в Замбии, Зимбабве, Кении, на Коморских Островах, в Лесото, Малави, Мозамбике, Намибии, Руанде, Свазиленде, Уганде, Эритрее и Эфиопии. Из 13 рассмотренных отчетов в 10 были определены недостатки, касающиеся одобрения комитетом по этике, в 9 — в расчетах размера выборки, в 6 — недостатки, касающиеся методов формирования выборки, в 12 — касающиеся обучения, в 13 — структуры контроля и в 11 — анализа данных.

**Вывод:**

Мы порекомендовали улучшить документацию по плановой и дополнительной вакцинации с помощью домашних карт вакцинации или иных записей. Для исследований, проведенных после дополнительной вакцинации, необходим стандартный протокол. Наконец, мы рекомендуем разработать стандарты для шаблонов отчета и для процедуры технического анализа протоколов и отчетов. Эти действия гарантируют, что результаты исследований охвата вакцинацией являются точными, сопоставимыми, надежными и ценными для улучшения программы.

## Introduction

Four types of surveys are commonly used to estimate vaccination coverage in developing countries: demographic and health surveys, multiple indicator cluster surveys, Expanded Programme on Immunization cluster surveys and surveys based on lot quality assurance sampling.^1^^,^^2^ Expanded Programme on Immunization cluster surveys have been used to assess coverage in supplementary measles or measles–rubella immunization activities. These surveys often include some evaluation of routine immunization, the communication strategies that have been used and the reasons for non-vaccination.^3^^,^^4^

Supplementary measles immunization activities are designed to ensure high population immunity against measles in areas that have yet to reach high levels of routine coverage with two doses of vaccine.^5^ Typically, nationwide catch-up activities, designed to eliminate susceptibility to measles in the general population, precede follow-up rounds of supplementary immunization. The follow-up rounds are generally conducted nationwide every 2–4 years and generally target children aged 9–59 months. They are designed to eliminate any measles susceptibility – especially in children born since the last round of supplementary immunization activities – and to protect those children who remain susceptible after receiving one dose of measles vaccine. Countries are encouraged to continue supplementary measles immunization until they reach and sustain 93–95% coverage with two doses of vaccine via routine immunization.

In monitoring the progress being made towards measles elimination, population-based surveys that incorporate probability sampling in all stages of sample selection and apply strict measures to minimize bias^1^ can provide accurate and reliable estimates of immunization coverage.^4^ Surveys of the coverage achieved after supplementary immunization activities may be hampered by problems in (i) obtaining the necessary funding far enough in advance, (ii) managing a logistical operation of considerable size that may be designed to measure the coverage achieved by the supplementary activities, by the routine immunization services, or by both the routine and supplementary interventions, and (iii) obtaining reasonably accurate subnational coverage estimates. The results of such surveys may be perceived as biased if the surveys are not conducted by organizations that are considered to be independent of the immunization programmes that are under scrutiny. Here we assess the methods used to evaluate and report coverage, identify quality concerns and provide recommendations for improvement.

## Methods

We reviewed the results of 13 coverage surveys conducted in 2012–2013, following 16 programmes of supplementary measles immunization. Either monovalent measles vaccine or measles–rubella vaccine had been administered. At least one other child health intervention – e.g. the distribution of oral poliomyelitis vaccine, tetanus toxoid vaccine, vitamin A or an anthelminthic drug – was delivered in conjunction with 15 (94%) of the programmes. We investigated the organization(s) undertaking each survey, survey design, sample size, the numbers of study clusters and children per study cluster, recording of immunizations and methods of analysis. We documented sampling methods at the level of clusters, households and individual children. We also assessed the length of training for field teams at national and regional levels, the composition of teams and the supervision provided.

The survey methods were compared with those recommended by WHO in 2005.^6^

## Results

All six rounds of supplementary measles immunization conducted in 2012 and nine of the 10 conducted in 2013 were followed by a coverage survey. At the time of our review, reports on only 13 of the 15 coverage surveys were available, since reports on two of the coverage surveys conducted in 2013 were pending. The surveys included in this review were performed in Comoros, Eritrea, Ethiopia, Kenya, Lesotho, Malawi, Mozambique, Namibia, Rwanda, Swaziland, Uganda, Zambia and Zimbabwe.

According to unpublished documents submitted to WHO, five (38%) of the 13 surveys were led by a local consultant or consultant firm, one (8%) by a national research institute – with technical support from consultants from several international organizations, four (31%) by WHO’s office in Harare, Zimbabwe, and two (15%) by international WHO consultants. Eleven (85%) of the 13 reported surveys had used either finger marks or immunization cards to identify children who had been vaccinated during the preceding round of supplementary measles immunization activities. Where cards had been used (in four of the surveys), data were available for a median of 69% (range: 45–100%) of the eligible children. Finger marks were used in seven surveys. In these surveys, data were available for a median of 48% (range: 3–100%) of the eligible children.

In nine (69%) of the 13 surveys reported, the supplementary immunization-coverage estimate based on the survey results was lower than that based on the corresponding, routinely collected administrative data. Based on finger marks, immunization cards or the recall of members of the study households, the survey results indicated a median coverage of 93% (range: 81–98%). However, the corresponding value based only on finger marks or cards was only 62% (range: 3–91%).

Eight (62%) of the 13 surveys reported had measured the achievement of full immunization with all routine vaccines by 11 months of age. In these eight surveys, an estimated median of 78% (range: 64–95%) of children aged 12–23 months were found to be fully immunized. The reports on six (75%) of these eight surveys described the method that had been used to document routine child immunization. The estimated median full routine vaccination coverage by 11 months of age – based only on the data held on immunization cards – was 69% (range: 63–77%). One country measured the achievement of full routine measles immunization by 23 months of age – as assessed in children aged 24–35 months. Here, routine vaccination coverage by 23 months of age – based only on the data held on immunization cards – was 86%. Seven of nine surveys that assessed routine immunization services also included coverage of tetanus vaccinations in women of childbearing age.

Table 1 shows selected results from our review. Only four of the reports we investigated provided details of the minimum sample size required per sampling area, as derived from sample size calculations. Most sample sizes were variations of a traditional design that involves 30 clusters and seven children per cluster. Although six of the reports provided information about the sampling frame, the originating year of the sampling frame and the methods used to adjust the data to the estimated population size at the time of the survey were not discussed in any of these reports. According to the reports we investigated, only one of the surveys reported any training at subnational level. Smaller size countries may not need training at subnational level (i.e. all survey teams are trained at national level); however, this should be discussed as part of survey protocols and reports. None of the reports included any information about the quality or consistency of supervision at subnational level. Although WHO recommends that inexperienced interviewers and supervisors be trained for at least 2–3 days,^6^ five (50%) of the surveys for which the relevant information was available reportedly included training for no more than 2 days. Another concern was the lack of detail in the reports on how the initial or index study household and subsequent households were selected within the study clusters. For example, it was generally unclear how random starting points were selected, how and if clusters were subdivided, how the population size per cluster subdivision was kept similar, how many household lists had to be obtained or generated and how such lists were obtained or generated. At least two of the surveys that we investigated had spun a bottle or pen, then selected households among 10 houses according to the direction indicated. This practice is not consistent with WHO guidelines.

**Table 1:** Selected results from a review of vaccination coverage surveys conducted to evaluate supplementary measles immunization in eastern and southern Africa, 2012–2013

Survey characteristics	No. (%) of survey reports (*n* = 13)	Comments
**Survey focus**		
SIA coverage only	4 (31)	None
SIA coverage with child routine coverage	2 (15)	None
SIA coverage with child routine coverage and tetanus vaccine coverage in WCBA	7 (54)	None
**Survey type**		
Two-stage cluster survey	13 (100)	None
**Sampling level or domain**		
National	1 (8)	None
Region or province	5 (38)	One report stated that, due to an inadequate number of respondents, 13 regions were collapsed to seven strata during analysis
District, zone or county	6 (46)	None
Other	1 (8)	One survey combined several zones into domains
**No. of survey clusters**		
30	10 (77)	None
15, 20 or 40	3 (23)	None
**No. of children per cluster investigated**		
7	1 (8)	None
8	2 (15)	None
9	1 (8)	None
10	6 (46)	None
Other	2 (15)	Fixed number of households; all eligible children per household selected
Not given in report	1 (8)	None
**Assumptions for sample-size calculation provided**		
Coverage	9 (69)	None
Precision	9 (69)	None
Design effect	10 (77)	None
**Sampling frame provided**	7 (54)	Median originating year of sampling frame was 2007 (range: 2000–2010)
**Ethical clearance provided**	3 (23)	None
**Training level**^a^		
National	6 (46)	None
Subnational	1 (8)	None
Not given in report	6 (46)	None
**Method of selecting index household within cluster**		
Bottle or pen spun at central cluster location	5 (38)	Two surveys selected an index household among 10 houses counted in the selected direction; one reported that the first household was selected in the direction in which a spun pen pointed
Other	6 (46)	Six survey reports described use of a random starting point in a cluster – without mentioning use of pen or bottle. One report described how clusters with large numbers of houses were subdivided before one subdivision was used at random. Two surveys reportedly selected an index household randomly from a household list. One of these two surveys had generated a household list where none was already available
Not given in report	2 (15)	None
**Method of selecting households within cluster after index household**		
Next household	4 (31)	None
Random selection	4 (31)	One survey reportedly used systematic random sampling and two surveys reportedly used a sampling interval
Not given in report	5 (38)	None
**Children selected per study household**		
All eligible children	3 (23)	None
One child	2 (15)	None
Not given in report	8 (62)	None
**Method used to verify immunization**		
Finger marks	7 (54)	None
Immunization cards	4 (31)	None
Not given in report	2 (15)	None
**Confidence intervals provided**	7 (54)	None

SIA: supplementary immunization activities; WCBA: women of childbearing age.

^a^ Training length varied from 1 day to 5 days (median: 2). The relevant information was missing from the reports of three surveys. None of the reports gave the criteria used for deciding length of training or the training agendas to be followed.

Seven of the surveys we investigated had used finger marks to verify vaccination. This practice only makes sense if the survey takes place soon after the vaccination round, since finger marks soon fade. Only four surveys (31%) had been conducted within 1 month of vaccination and eight (62%) failed to report survey dates. 

Of the 13 reports, six (46%) provided information that allowed the number of field teams per supervisor to be calculated. Of those, three (50%) two (33%) and one (17%) described the use of one, two and four teams per supervisor, respectively. There were two interviewers per team in all but one survey, which used four interviewers per team. Just seven (54%) of the 13 reports we investigated provided details about the selection of clusters; six described cluster selection that was proportional to population size and one described cluster selection by square root allocation. Although nine (69%) of the 13 reports provided information about the software used for data management and analysis, only two provided more specific information about the analysis process, including the adjustment for cluster design and weighting.

## Discussion

Our review showed that coverage surveys have become a regular component of supplementary measles immunization activities in the countries studied. Most of the reviewed surveys included some investigation of routine immunization services. All of them used some variation of the two-stage cluster survey design recommended by WHO in guidelines published in 2005.^6^ However, sample size calculations were provided in less than one third of the survey reports we investigated. We found the potential for sampling bias related to uncertainty about the adequate updating of population data for sampling frames, the selection of index households in study clusters and the measures used to ensure the random selection of subsequent study households. The recommended generation and use of household lists in clusters,^1^ was only described in a small number of the survey reports and, even then, more detail about how such lists – and how many – were obtained or generated would have been useful. 

The timing of surveys and the methods of documenting the immunizations were other areas of concern. Delays in conducting coverage surveys might have allowed finger marks to fade, resulting in an increased dependence on recall and an increased risk of information bias. A culture of providing, using, updating and retaining high-quality home-based records, such as immunization cards, could ensure that coverage estimates – for both routine and supplementary immunizations – are based more on documented doses and less on recall. Another potential problem was the frequent lack of independence between those who conducted the coverage surveys and those who were responsible for implementing the immunizations. Training and supervision of the field teams often appeared inadequate. WHO recommends that a supervisor should not be responsible for more than two interview teams.^6^ Although most of the surveys we investigated used no more than two teams per supervisor, the corresponding survey reports provided too little detail about the length and contents of training for the teams – including the capacity needed at subnational level. None of the surveys described supervision that covered all levels of the immunization programme – i.e. from national level to cluster level. Ideally, any data analysis should follow a detailed plan that forms part of the survey protocol. Survey reports should include information not only about the software used for data analysis but also on how the software was used to adjust for cluster design, and whether weighting was used. Weighting becomes particularly important if all the eligible children in a fixed number of households per cluster are investigated, rather than a fixed number of children per cluster.^6^ Survey reports should also include a standard set of tables and figures representing a fixed minimum number of data elements, and full data sets should be available to other researchers for additional analyses, such as between-country comparisons. Finally, as recommended by WHO guidelines,^6^ ethical approval for coverage surveys should be obtained in each country.

Our review has some limitations. A retrospective assessment cannot determine whether, during a reported survey, certain methods were applied but not reported, reported but not applied or neither reported or applied. We made no attempt to seek clarification or further information from the authors of the reports we studied. We focused on surveys conducted after rounds of supplementary immunization activities and ignored other types of coverage survey – e.g. Expanded Programme on Immunization surveys of routine immunization activities or evaluations of outbreak response immunization activities. Our main limitation was the lack of detail or absence of relevant information in many of the reports. This often limited our ability to assess the design and implementation of a survey fully. For example, it was often impossible to determine whether the recommended standards for probability sampling^1^^,^^6^ had been met.

The participants at the consultative meeting in Zimbabwe that followed this review made recommendations on coverage survey methods. They called for further advocacy around appropriate advanced planning of coverage surveys – concurrent with campaign planning itself – and for the development of a standard protocol and report templates with the required level of detail. They recommended that full data sets from coverage surveys be made available to other researchers – to allow further analyses and comparisons across countries. A standardized technical review for protocols and reports, the development of criteria for selecting independent groups to conduct coverage surveys and further assessment of the appropriate duration and content of field training and supervision were also recommended. 

WHO’s guidelines on survey design, conduct and reporting are currently being updated, with the main aim of improving the quality of all vaccination coverage surveys – including those conducted after supplementary immunization activities. Organizations that are active in immunization programmes should ensure adherence to the current guidelines now and to the updated guidelines as soon as they become available. High-quality cluster surveys are considered a routine component in monitoring the progress of immunization systems, within the context of the Global Vaccine Action Plan.^7^ All supplementary measles or measles–rubella immunization activities should include a plan and budget for a coverage survey, as part of a general monitoring and evaluation plan.^8^ Although WHO will provide technical assistance – via its own staff or the engagement of external consultants – and sometimes financial support, it leaves the decision on whether or not to conduct a coverage survey and the organization of any such survey to its Member States. 

In addition to WHO’s guidelines, implementation of the recommendations from this review and the consultative meeting that followed it should help to ensure that the results of immunization coverage surveys are accurate, reliable and useful for programme improvement.
